# Local melatonin application induces cold tolerance in distant organs of *Citrullus lanatus* L. via long distance transport

**DOI:** 10.1038/srep40858

**Published:** 2017-01-19

**Authors:** Hao Li, Jingjing Chang, Junxian Zheng, Yuchuan Dong, Qiyan Liu, Xiaozhen Yang, Chunhua Wei, Yong Zhang, Jianxiang Ma, Xian Zhang

**Affiliations:** 1 College of Horticulture, Northwest A&F University, Taicheng Road 3, Yangling 712100, Shaanxi, P.R. China

## Abstract

Melatonin is a ubiquitous chemical substance that regulates plant growth and responses to stress. Several recent studies show that exogenous melatonin confers cold tolerance to plants; however, the underlying mechanisms remain largely unknown. Here, we report that melatonin application at optimal dose, either on the leaves or the roots, not only induced cold stress tolerance in the site of application, but also systemically induced cold tolerance in untreated distant parts. Foliar or rhizospheric treatment with melatonin increased the melatonin levels in untreated roots or leaves, respectively, under both normal and cold stress conditions, whereas rhizospheric melatonin treatment increased the melatonin exudation rates from the xylem. An increased accumulation of melatonin accompanied with an induction in antioxidant enzyme activity in distant untreated tissues alleviated cold-induced oxidative stress. In addition, RNA-seq analysis revealed that an abundance of cold defense-related genes involved in signal sensing and transduction, transcriptional regulation, protection and detoxification, and hormone signaling might mediate melatonin-induced cold tolerance. Taken together, our results suggest that melatonin can induce cold tolerance via long distance signaling, and such induction is associated with an enhanced antioxidant capacity and optimized defense gene expression. Such a mechanism can be greatly exploited to benefit the agricultural production.

Since plants cannot relocate, they have to face multiple biotic and abiotic stresses throughout their life cycle. Among these stresses, cold stress adversely affects plant growth and development, and thus is considered as one of the most important environmental hazards that limit the spatial distribution of plants and agricultural productivity^1^. Cold stress inhibits various plant physiological processes by directly altering multiple metabolic reactions, while indirectly, it induces other stresses including osmotic and oxidative stresses. To survive cold stress, plants have evolved intricate signaling networks that eventually help plants to adapt to the changing temperatures by optimizing cellular activities. Molecular receptors localized on plant cell membranes can sense any changes in temperatures and generate secondary signals to activate different transcriptional regulators via activation of phosphoprotein kinases, which eventually induce the expression of major stress responsive genes and proteins to prevent and/or repair cold-induced damage^2^,^3^,^4^. Moreover, accumulating data support a crucial role of plant hormones in governing signal events in the cold stress response^5^.

At an organismal level, certain plant tissues, either shoot or root, are not isolated, rather communicate with each other to fine-tune regulation of growth, development, and responses to stresses. In particular, shoot to root communication or vice-versa improves plant survival during unfavorable environmental conditions. Long-distance signals that are also involved in the stress response play critical roles in such communication between different tissues. The roots of many plants, for example, produce more ABA in response to soil drought. ABA is then transported to the leaves, where it triggers stomatal closure to minimize water loss from the leaves^6^. Methyl salicylate functions as a critical mobile signal, which is elicited at the primary site of pathogen attack but acts on distant tissues to induce ‘systemic acquired resistance’^7^. Immovable plant growth regulators (such as brassinosteroids) can induce tolerance to abiotic or biotic stresses in distant organs by propagating secondary signals such as hydrogen peroxide (H_2_O_2_)^8^. Owing the diversity and versatility of plant signaling molecules, elucidation of various long distance signals that potentially mediate plant tolerance to cold stress has appeared as an important research avenue in plant science.

Melatonin (*N*-acetyl-5-methoxytryptamine) is a highly conserved molecule that is ubiquitously present in living organisms ranging from bacteria to mammals^9^. About two decades ago, melatonin was identified in vascular plants^10^,^11^. Melatonin has been shown to have important regulatory roles in plant defense against biotic and abiotic stresses, such as, extreme temperatures, excess copper, salinity, and drought^12^,^13^. Melatonin as an antioxidant, protects cells from oxidative/nitrosative stress by scavenging toxic free radicals^14^. Nonetheless, it also stimulates plant antioxidant systems^14^. Recently, several studies have shown that exogenous melatonin at optimal concentrations can enhance cold tolerance in a range of plant species including *Arabidopsis, Triticum aestivuml*, and *Citrullus lanatus*^15^,^16^,^17^,^18^. Notably, melatonin-induced enhancement in cold tolerance was closely associated with the regulation of genes involved in stress response and signal transduction.

Melatonin is synthesized from tryptophan through enzymatic conversion and has similar structural moieties to natural auxin, and thus likely to transport over long distance from a site of synthesis to a site of function in distant tissues^19^. Melatonin contents in leaves of water hyacinths can be elevated by exogenous melatonin application to growth media^20^. Moreover, melatonin levels in both roots and cotyledons are induced following exposure of sunflower seedlings to NaCl stress, indicating potential involvement of melatonin in long distance signaling from roots to cotyledons during salt stress^21^. Nonetheless, whether melatonin can be transported from leaves to roots in response to stress remains elusive. Our previous study revealed that exogenous melatonin application on roots is capable to alleviate photooxidative stress in leaves of cucumber^22^. However, direct evidence for melatonin as a mobile signal is still lacking.

The watermelon (*Citrullus lanatus* L.), is one of the most economically important crops in the world, but highly sensitive to low temperatures^23^. Here, we analyzed the effects of foliar and rhizospheric melatonin pretreatment on the cold stress tolerance in untreated leaves and roots, respectively. We determined melatonin content of leaves, roots, and xylem sap, as well as the melatonin exudation rate from the xylem under both normal and cold stress conditions. Additionally, we analyzed the effects of melatonin on the antioxidant systems and defense gene networks that respond to cold stress, using high-throughput mRNA sequencing analysis. Our results suggest that melatonin is a mobile signal, capable of inducing cold tolerance in both local and distant organs. This induction is closely associated with enhanced antioxidant capacity and a defined set of cold response genes. Such a mechanism could be greatly exploited to benefit the agricultural production especially in the season of low temperature.

## Results

### Melatonin confers cold tolerance to both local and distant organs

**Figure 1:**
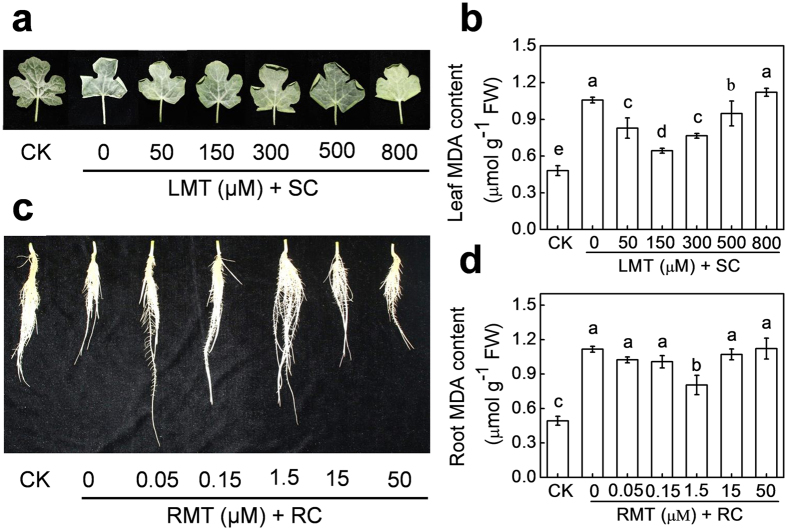
Effects of melatonin on leaf and root tolerance to aerial and rhizospheric cold stress, respectively. (**a**,**b**) Leaves of watermelon (*Citrullus lanatus* L.) seedlings at the four-leaf stage were pre-treated with melatonin at 0, 50, 150, 300, 500 or 800 μM (LMT) for three times (once a day). Subsequently, the plants were exposed to aerial cold stress at 4 °C (SC) for 72 h. (**c**,**d**) Roots of watermelon seedlings at the four-leaf stage were pre-treated with melatonin at 0, 0.05, 0.15, 1.5, 15 or 50 μM (RMT). Subsequently, the plants were exposed to rhizospheric cold stress at 10 °C (RC) for 72 h. In (**a,b**), leaf phenotypes and leaf MDA contents were monitored to assess changes in the cold tolerance of leaves. In (**c**,**d**), the root phenotypes and root MDA contents were monitored to assess changes in the cold tolerance of roots. Data of MDA contents show the means of three replicates (±SD). Means denoted with the same letter did not significantly differ at *P* < 0.05.

As shown in Fig. 1, application of appropriate concentrations of melatonin on leaves (LMT) alleviated aerial cold (SC)-induced wilting of blade edges and lipid peroxidation as evident from malondialdehyde (MDA) content. Similarly, melatonin application at appropriate concentrations on roots (RMT) decreased rhizospheric cold (RC)-induced root growth inhibition and MDA content. The most effective melatonin concentrations that conferred cold tolerance were 150 μM and 1.5 μM for leaves and roots, respectively. MDA content of leaves treated with 150 μM melatonin was 39.2% lower compared to control leaves after exposure to SC stress. Similarly, MDA content of roots treated with 1.5 μM melatonin was 27.9% lower compared to control roots after exposure to RC stress. However, both higher and lower concentrations of melatonin other than the optimum either attenuated or compromised the protective effect of melatonin against cold stress.

**Figure 2:**
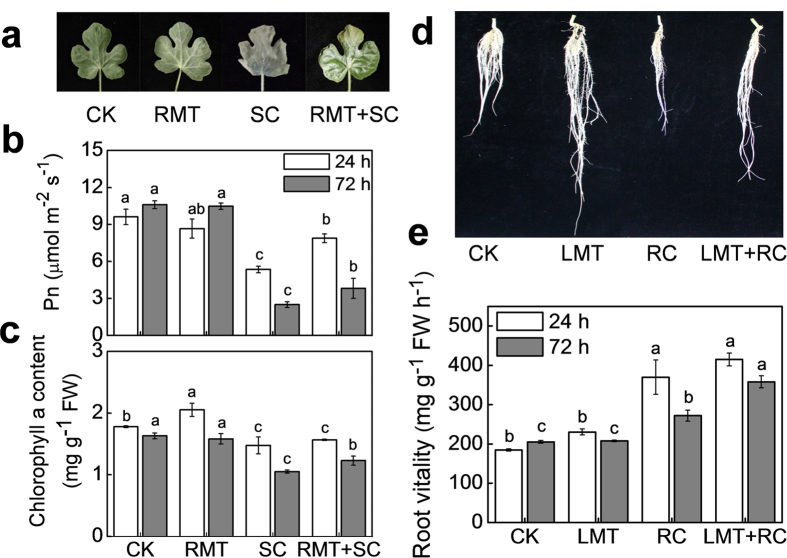
Enhanced tolerance to cold stress in untreated distant leaves and roots was induced by local melatonin application. (**a**–**c**) Roots of watermelon seedlings at the four-leaf stage were pre-treated with 1.5 μM melatonin (RMT) before the seedlings were exposed to aerial cold stress at 4 °C (SC) for 72 h. (**d**,**e**) Leaves of watermelon seedlings at the four-leaf stage were pre-treated with 150 μM melatonin (LMT) for three times (once a day). Subsequently, the seedlings were exposed to rhizospheric cold stress at 10 °C (RC) for 72 h. In (**a**–**c**), leaf phenotypes, Pn, and chlorophyll a contents were monitored to assess changes in the cold tolerance of leaves. In (**d**,**e**), the root phenotypes and root vitality were monitored to assess changes in the cold tolerance of roots. Data of Pn are the means of six replicates (±SD). Data of chlorophyll a content and root vitality are the means of three replicates (±SD). Means denoted with the same letter did not significantly differ at *P* < 0.05.

To determine whether melatonin treatment induced stress tolerance in leaves or roots system-wide, we treated roots with 1.5 μM melatonin or leaves with 150 μM and then subjected the plants to SC or RC stress, respectively. As shown in Fig. 2, SC stress caused leaf wilting and reduced net photosynthetic rate (Pn) and chlorophyll a (Chl a) content, while RC stress inhibited root growth and induced root vitality. However, RMT treatment alleviated leaf wilting and reduced Pn and Chl a content caused by SC at both 24 h and 72 h. Similarly, LMT treatment alleviated RC-caused inhibition of root growth, but promoted RC-induced root vitality at 72 h. Pn and Chl a content in plants with RMT treatment were increased by 52.2% and 17.1% respectively compared to control after SC treatment for 72 h. Root vitality in plants with LMT treatment was increased by 33.3% compared to control after RC treatment for 72 h. These results clearly indicate that in addition to stress ameliorative effect of melatonin on site of application, local application of melatonin on leaves or root can induce cold tolerance in distant roots or leaves, respectively.

### Changes in melatonin contents and exudation rate from the xylem as influenced by cold stress and exogenous melatonin treatment

**Figure 3:**
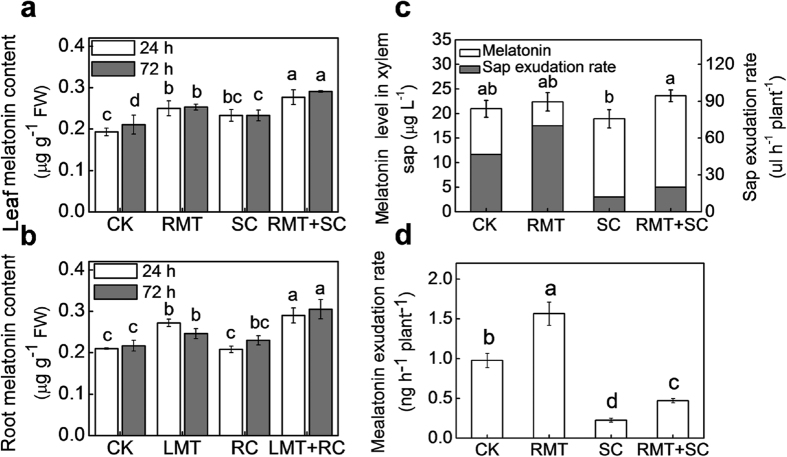
Analysis of melatonin levels in leaves and roots, and melatonin exudation rates from the xylem. Watermelon seedlings at the four-leaf stage were treated as described in Fig. 2, and the xylem sap was collected at 24 h after exposure to aerial cold stress. (**a**) Changes in melatonin contents of leaves after rhizospheric melatonin treatment and, or aerial cold stress. (**b**) Changes in melatonin contents of roots after foliar melatonin treatment and, or rhizospheric cold stress. (**c**,**d**) Changes in melatonin transport with in xylem after rhizospheric melatonin treatment and, or aerial cold stress. Data are the means of three replicates (±SD). Means denoted with the same letter did not significantly differ at *P* < 0.05.

Melatonin contents in leaves and roots remained virtually unchanged by SC or RC stress alone. However, the melatonin content of leaves in plants with RMT treatment significantly increased under normal and especially under SC stress conditions (Fig. 3a). Similarly, root melatonin content in plants subjected to LMT treatment significantly increased under normal and especially under RC stress conditions (Fig. 3b). To further evaluate whether melatonin was transported from melatonin-treated roots to untreated leaves via vascular bundles, we analyzed melatonin exudation rates from the xylem after RMT and, or SC treatments. As shown in Fig. 3c, melatonin levels in xylem sap significantly decreased due to SC stress in control plants, but not in RMT treated plants. While, the xylem sap exudation rate was increased by RMT treatment, but was decreased by SC treatment. Finally, melatonin exudation rates from the xylem of RMT treated plants were increased by 60.2% and 104.3% under normal (CK) and SC stress conditions, respectively, compared to control plants (Fig. 3d).

### Melatonin alleviates cold-caused oxidative stress in untreated distant tissues

**Figure 4:**
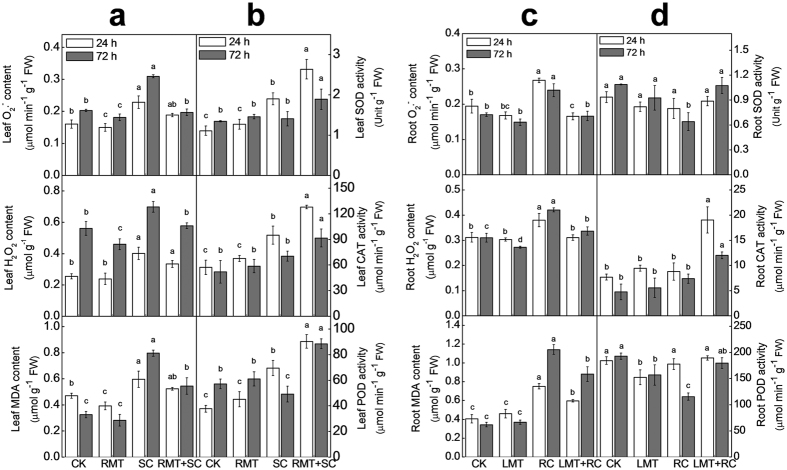
Effects of rhizospheric or foliar melatonin application on oxidative stress and antioxidant enzyme activities in untreated distant organs after cold stress. Watermelon seedlings at the four-leaf stage were treated as described for Fig. 2, and the samples were harvested at 24 h and 72 h after either aerial or rhizospheric cold stress. (**a** and **b**) Oxidative stress and antioxidant enzyme activities in leaves after rhizospheric melatonin treatment and, or aerial cold stress. (**c** and **d**) Oxidative stress and antioxidant enzyme activities in roots after foliar melatonin treatment and, or rhizospheric cold stress. Data are means of three replicates (±SD). Means denoted with the same letter did not significantly differ at *P* < 0.05.

SC and RC treatment induced the accumulation of reactive oxygen species (ROS, including O_2_·^−^ and H_2_O_2_) and subsequently increased MDA in leaves and roots, respectively (Fig. 4). However, RMT and LMT treatment alleviated cold-induced increases in ROS and MDA in untreated leaves and roots, respectively. Antioxidant systems (such as antioxidant enzymes and non-enzymatic oxidants) play critical roles in the defense against oxidative stress. Under normal growth conditions, the activities of superoxide dismutase (SOD), catalase (CAT), and peroxidase (POD) in leaves and roots were virtually unchanged in plants with melatonin treatment on roots and leaves, respectively. Following SC stress, the activities of these enzymes in leaves increased at 24 h and then decreased to initial or lower levels at 72 h. However, after RC stress, the activities of these enzymes in roots remained unchanged at 24 h and decreased at 72 h. Interestingly, RMT treatment with SC increased almost all tested antioxidant enzyme activities in leaves at both 24 h and 72 h, compared to those in SC alone. Similarly, LMT treatment on RC-stressed plants increased activities of all those antioxidant enzyme activities in roots at both 24 h and 72 h, compared to those in RC alone-stressed plants.

### Melatonin regulates cold defense genes in leaves

**Figure 5:**
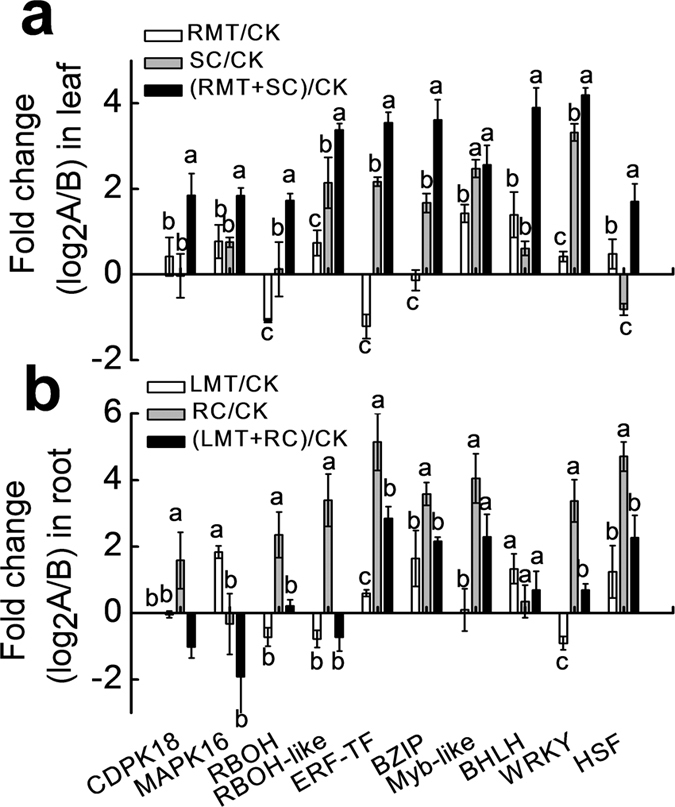
Expression analysis of cold defense-related genes via qRT-PCR in untreated distant organs by local melatonin application to roots or leaves. Watermelon seedlings at the four-leaf stage were treated as described in Fig. 2, and the samples were harvested at 24 h after aerial or rhizospheric cold stress. (**a**) Relative expression of genes in leaves after rhizospheric melatonin treatment (1.5 μM) and, or aerial cold stress (4 °C). (**b**) Relative expression of genes in roots after foliar melatonin treatment (150 μM) and, or rhizospheric cold stress (10 °C). mRNA expression was normalized via *β-actin* levels. All reactions for the qRT-PCR were repeated three times for each sample. Data are means of three replicates (±SD). Means denoted with the same letter did not significantly differ at *P* < 0.05.

To examine the involvement of cold defense-related genes in the melatonin-mediated cold tolerance in distant leaves or roots, we analyzed the changes in expression of critical genes in the cold defense via qRT-PCR. These genes are involved in signaling cascades including *calcium dependent protein kinase (CDPK*) *18, mitogen-activated protein kinase (MAPK*) *16, respiratory burst oxidase homologue (RBOH*), and *RBOH*-*like* and transcription regulation including *ethylene-responsive transcription factor (ERF*-*TF*), *basic leucine zipper domain (BZIP*), *Myb*-*like, basic helix-loop-helix (BHLH*), *WRKY*, and *heat stress transcription factor (HSF*). RMT treatment alone slightly up-regulated the expression levels of *CDPK18, MAPK16, RBOH*-*like, Myb*-*like, BHLH, WRKY*, and *HSF* in leaves, while LMT treatment alone slightly up-regulated the transcription of *MAPK16, ERF-TF, BZIP, BHLH*, and *HSF* in roots (Fig. 5). After exposure of watermelon plants to SC or RC stress, most of these genes in leaves or roots were up-regulated, respectively, compared to control. Intriguingly, RMT treatment further increased expression of these genes in leaves under SC stress resulting in higher transcriptional levels of these genes in RMT + SC treatment compared to SC treatment alone. However, LMT treatment repressed the expression of most tested genes in roots under RC stress, resulting in lower transcriptional levels of these genes for LMT + RC treatment compared to RC treatment alone. These results suggest a potential involvement of these cold-responsive genes in RMT-induced cold tolerance of leaves, but not in LMT-induced cold tolerance of roots.

**Figure 6:**
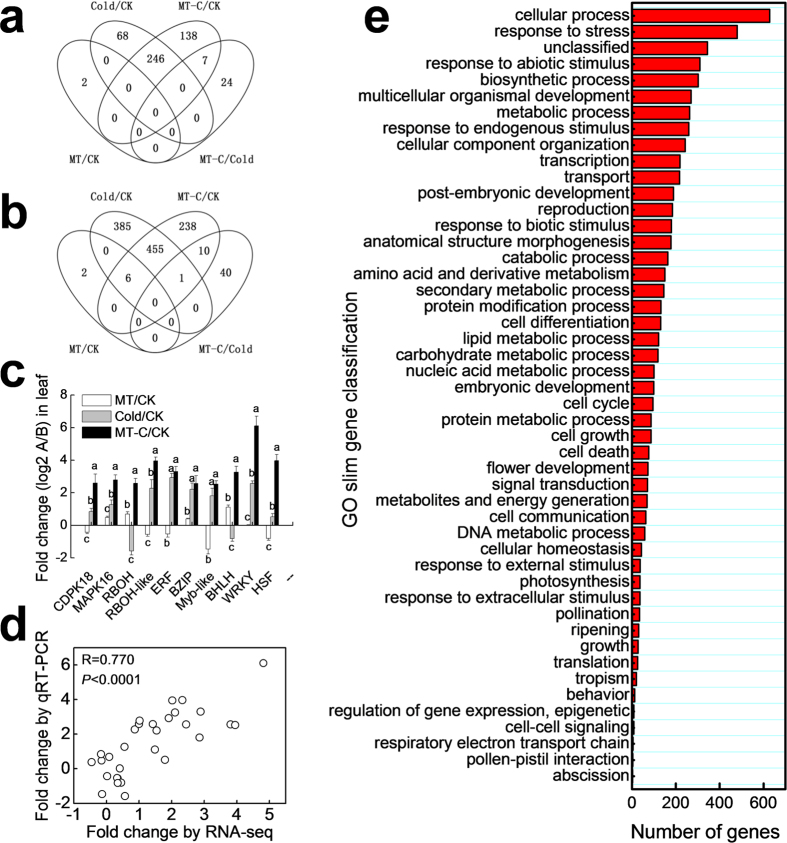
Analysis of differentially expressed genes induced by melatonin and, or cold stress based on mRNA-seq. Watermelon seedlings (whole plants) at the four-leaf stage were exposed to cold stress at 4 ^o^C either with or without melatonin pretreatment (150 μM) on the leaves. Leaf samples were taken at 6 h and high-throughput mRNA sequencing was performed using two biological replicates for each treatment. Venn diagrams of (**a**) up-regulated and (**b**) down-regulated genes. (**c**) Expression analysis of cold defense-related genes via qRT-PCR. (**d**) Correlation analysis of 10 differentially expressed genes, such as, *CDPK 18, MAPK 16, RBOH, RBOH*-*like, ERF*-*TF, BZIP, Myb*-*like, BHLH, WRKY*, and *HSF* between RNA-seq and qRT-PCR analyses. (**e**) Gene Ontology classification for differentially expressed genes based on their involvement in various biological processes. In (**c**), data are means of three replicates (±SD). Means denoted with the same letter did not significantly differ at *P* < 0.05.

We then performed RNA-seq analysis of leaves treated with distilled water (CK), melatonin (MT), cold (Cold), and melatonin + cold (MT-C). A total of 290748960 raw reads from all samples were obtained (Supplemental Table S2). After removal of rRNAs, tRNAs, snRNAs, and snoRNAs, a total of 5327079/5914071, 5635109/6581846, 6219825/5085935, and 5409138/6272887 mRNA sequences remained for CK-1/2, MT-1/2, Cold-1/2, and MT-C-1/2, respectively. Compared to the control (CK), MT, Cold, and MT-C treatments differentially changed the transcription of a total of 10 genes (2 up-regulated, 8 down-regulated), 1,161 genes (314 up-regulated and 847 down-regulated), and 1,101 genes (391 up-regulated and 710 down-regulated), respectively (Fig. 6). Compared to cold stress alone, a total of 31 and 51 genes were significantly up-regulated and down-regulated by MT-C, respectively. Additionally, we analyzed the changes in expression of critical genes involved in signaling cascades including *CDPK 18, MAPK 16, RBOH*, and *RBOH*-*like* and transcription regulation including *ERF*-*TF, BZIP, Myb*-*like, BHLH, WRKY*, and *HSF* in the cold defense via qRT-PCR. Across all treatments, the RNA-seq results between the two biological replicates were strongly correlated (Supplemental Figure S1) and the results of RNA-seq analysis were similar compared to those obtained via qRT-PCR (R = 0.77; *P* < 0.0001), indicating that the changes in expression detected via RNA-seq were accurate. We also subjected the differentially expressed genes to Gene Ontology (GO) classification based on their involvement in the Cucurbit Genomics Database (http://www.icugi.org) with watermelon 97103 v1. As categories, cellular process, response to stress, unclassified, and response to abiotic stimulus were the most abundant GO terms induced by melatonin and, or cold stress.

### Transcriptome profiles of signal receptor- and secondary signaling-related genes

Melatonin treatment alone had negligible effects on the transcription of most signal receptor- and secondary signaling-related genes (Table 1). Following exposure to cold stress, the transcription of 12 receptor genes significantly decreased, however, these decreases could be alleviated by melatonin pretreatment. Moreover, the transcription of three receptor genes (encoding Receptor-like kinase, Receptor protein kinase-like protein, and Lectin receptor kinase 1) and four receptor genes (encoding Receptor protein kinase-like protein, Leucine-rich repeat (LRR) receptor-like protein kinase, LRR receptor-like tyrosine-protein kinase, and G-type lectin S-receptor-like serine/threonine-protein kinase) were significantly up- and down-regulated by MT-C treatment, respectively, but not in cold stress alone.

Exposure to cold stress decreased expression of calcium signaling-related genes (including *CDPK 25, Calmodulin binding protein*, and *Sodium-calcium exchanger 3*), but these decreases were alleviated by melatonin pretreatment. Moreover, MT-C treatment but not cold stress alone induced transcription of *Calcium-dependent membrane targeting, Calmodulin-binding protein, Plasma membrane calcium-transporting ATPase 3*, and *Calcium/proton exchanger*. The gene *Respiratory burst oxidase-like protein* may be involved in encoding NADPH oxidase and generating H_2_O_2_, and was up-regulated by MT-C treatment only. For genes involved in inositol 1, 4, 5-trisphosphate signaling, melatonin pretreatment alleviated cold-induced up-regulation of *Acyl-protein thioesterase 2* and *Membrane transporter D1 (Cla009398*) and down-regulation of *COBRA-like protein* and *Membrane transporter D1 (Cla015945*), also down-regulating the expression of *1-phosphatidylinositol-4* and *5-bisphosphate phosphodiesterase Phospholipase A1* under cold stress.

### Transcriptome profiles of transcription factors and protective genes

Melatonin treatment by itself had negligible effects on the transcription of all transcription factors (TFs) and protective genes (Table 2). Cold stress alone down-regulated the transcription of 15 TFs (including 1 *BZIP* TFs, 6 *MYB* TFs, 5 *MYC/BHLH* TFs, 1 *WRKY* TFs, and 2 *HSF* TFs) and 12 protective genes (including 1, 8, 2, and 1 genes encoding LEA-HRGP, HSPs, Peroxidase, and Lipoxygenase). Interestingly, melatonin pretreatment alleviated cold-induced down-regulation of most TFs and protective genes. However, cold stress increased the transcription of three TFs (*MYB-like, MYB 5*, and *WRKY 4*) and two protective genes (*DnaJ* and *Peroxidase*). Melatonin and cold combined led to an up-regulation of the transcription of 11 TFs (including 1 *BZIP*, 4 *MYB*, 3 *MYC*, 1 *WRKY*, and 2 *HSF*) and 3 protective genes including (*HSP20, DnaJ*, and *Lipoxygenase*). Finally, transcription of five genes including *BZIP, BHLH, Peroxidase, Peroxidase a*, and *Lipoxygenase* were significantly induced by MT-C treatment in comparison to Cold treatment.

### Transcriptome profiles of hormone signaling-related genes

Under optimal growth temperatures, melatonin treatment did not alter the expression of genes involved in various hormonal pathways (Table 3). Cold stress significantly suppressed five *ERF transcription factors (Cla013573, Cla022212, Cla016785, Cla014051*, and *Cla022648*) of the ethylene (ET) pathway. However, melatonin pretreatment alleviated cold-induced down-regulation of these genes and up-regulated other *ERF transcription factors (Cla002237, Cla021069, Cla021070*, and *Cla017389*). For the gibberellin (GA) pathway, *Limonene synthase* and *GATA transcription factor 8* were up- and down-regulated in cold treatment, respectively, but not in the MT-C treatment. However, *D-limonene synthase, Gibberellin 2-oxidase*, and *Gibberellin-regulated family protein* were only significantly up-regulated following MT-C treatment. For the auxin pathway, *Auxin transporter-like protein 1, Auxin response factor (Cla009105, Cla015002*), *Iaa-amino acid hydrolase 11*, and *Auxin responsive protein (Cla014809, Cla019806*) were suppressed by cold treatment only, while *Auxin-induced SAUR-like protein (Cla015856, Cla016616*) and *IAA-amino acid hydrolase* were up- and down-regulated by MT-C treatment only, respectively. For the abscisic acid (ABA) pathway, *Abscisic acid receptor PYL8* was significantly suppressed by MT-C treatment, but not by cold stress alone. For the cytokinin pathway, *Cyclin D* and *Cytokinin oxidase/dehydrogenase* were decreased by cold and MT-C treatment, respectively. For the jasmonic acid (JA) pathway, *Protein TIFY 7* was up-regulated by both cold and especially by MT-C treatment. However, *Jasmonate ZIM-domain protein 3* was significantly up-regulated by MT-C only. Finally, compared to cold treatment, melatonin and cold combined up-regulated the expression levels of *ERF transcription factors (Cla021070, Cla022648*), *Gibberellin 2-oxidase*, and *Cyclin D3-1*.

## Discussion

Over the past several years, numerous studies have reported that melatonin plays vital roles in regulating plant defense against a wide range of biotic and abiotic stresses^13^. In agreement with previous studies^15^,^17^,^18^, we found that pretreatment with melatonin alleviated cold-induced damage of leaves and roots, which was precisely dose dependent (Fig. 1). Notably, with both higher and lower melatonin concentrations (except for the optimal concentration of 150 μM for leaves and 1.5 μM for roots), the protective effect of melatonin against cold stress was attenuated or even disappeared completely. Our previous study indicated that melatonin treatment enhanced the tolerance to Methyl viologen-activated photooxidative stress and this phenomenon remains valid not only for directly treated tissues, but also for untreated distant tissues^22^. Similarly, application of melatonin onto roots or leaves led to a systemic induction of cold tolerance in both untreated leaves and roots (Fig. 2). These results confirm that melatonin is capable of inducing cold tolerance in both local and distant systemic organs.

**Figure 7:**
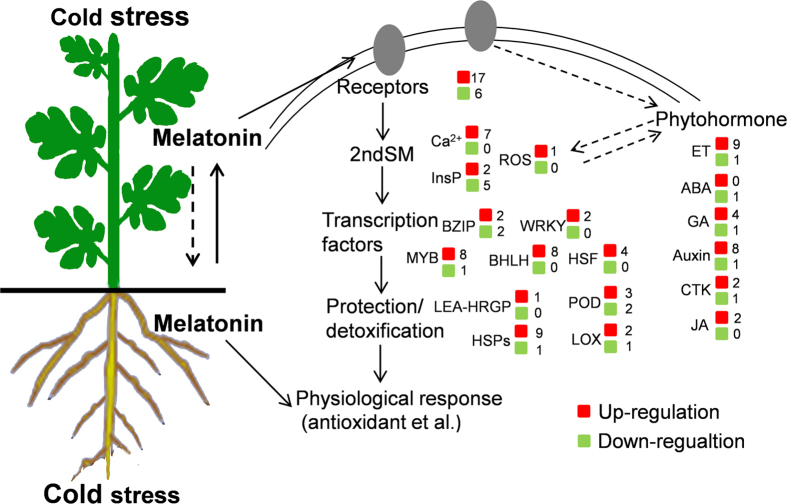
A model depicting local melatonin application-induced cold tolerance in local and distant tissues and the involvement of defense-related gene networks.

Induction of a systemic tolerance depends on the spread of systemic signals, initiated in stimulated tissue, translocated via vascular tissue to distal portions of the plant, where they are perceived in systemic tissues^24^,^25^. Many biologically active molecules have been identified as mobile signals in the sap of phloem and xylem^26^,^27^. Nearly all stress factors increase melatonin biosynthesis in investigated plants^13^,^14^, and several studies have suggested melatonin to be a novel long-distance signal distributed through the vascular bundle^20^,^21^,^28^. In our present study, we observed that application of melatonin on leaves and roots increased the melatonin levels in untreated distant roots and leaves, respectively, under normal and especially under cold stress conditions (Fig. 3). Furthermore, detection of melatonin in the xylem sap provides direct evidence for vascular transport of melatonin. The melatonin exudation rate from the xylem was significantly increased via rhizospheric melatonin treatment. Taken together, our results indicate that melatonin might be translocated from melatonin-treated roots (having increased melatonin level) to vascular bundles and then transported to untreated leaves via the xylem, thereby inducing cold tolerance in leaves (Fig. 7). However, due to technical limitations in collecting the phloem sap from four-leaf stage watermelon seedlings, we are unable to confirm, whether melatonin was also transported from leaves to roots via the phloem.

The primary role of melatonin in stress mitigation is considered to be as a broad-spectrum antioxidant^14^,^29^. Both aerial and rhizospheric cold induced the accumulation of ROS such as O_2_·^–^ and H_2_O_2_, subsequently damaging membranes through lipid peroxidation in leaves and roots, respectively^30^ (Fig. 4). O_2_·^–^ is easily converted to H_2_O_2_ by the catalysis of SOD, while H_2_O_2_ is scavenged via CAT and an AsA-GSH cycle^31^. Melatonin treatment on roots or leaves induced activities of antioxidant enzymes such as SOD, CAT, and POD and alleviated cold-caused oxidative stress in untreated tissues, indicating that melatonin-induced cold tolerance in distant tissues is closely associated with the enhancement in antioxidant system.

Except for directly reducing the rates of biochemical reactions, cold stress also indirectly affects membrane fluidity, cellular metabolism, as well as protein and nucleic acid conformation via the reprogramming of gene expression. Recent studies have revealed that melatonin can activate defense systems by regulating the expression of cold-responsive genes, such as *ZAT10, ZAT12, CBFs, COR15*, and *CAMTA1*^15^,^16^. In our study, we found that exogenous melatonin promoted cold-induced up-regulation of a set of regulatory genes involved in signal transduction and transcriptional regulation in leaves, but not in roots (Fig. 5). This is possibly attributed to the divergence in gene expression between leaves and roots during cold acclimation, since 86% of cold-induced genes are not shared between leaves and roots^32^. Thus, melatonin might play important roles for regulating gene networks of leaves during cold stress.

Generally, when plants suffer from cold stress, the extracellular stress signal is first perceived by the membrane receptors^1^,^2^,^3^. Recent studies have reported that higher plants have Receptor-like kinases (RLKs) that are involved in perception, amplification, and transmission of environmental stimuli via signaling cascades that modulate gene expressions, protein activation and finally elicit adjustment of the cellular milieu^33^. The LRR receptor kinases are one of the largest and most renowned classes of RLKs and they play important roles for positive regulations of cold stress tolerance^34^,^35^. The perception of cold stimuli by receptors can activate large and complex intracellular signaling cascades, leading to the generation of secondary signal molecules including Ca^2+^, ROS, and inositol 1, 4, 5-trisphosphate^36^. Here, we found that pretreatment with melatonin altered the expression of a set of genes that are involved in encoding signal receptors (i.e. *RLKs* and *LRR receptor kinases*) and secondary signaling such as Ca^2+^ (i.e. *CDPK 25, Calcium-dependent membrane targeting*, and *Calmodulin-binding protein*) and H_2_O_2_ (i.e. *Respiratory burst oxidase-like protein*) during cold stress (Table 1). These results suggest that melatonin may be involved in the sensing of the cold signal and subsequent intracellular signal transduction. However, the receptors and mechanisms those enable plants to perceive temperature remain largely elusive. In addition, an increase in cytosolic calcium boosts H_2_O_2_ generation by activating NADPH oxidase^37^,^38^ and inhibiting calmodulin (CaM, a ubiquitous calcium-binding protein) leading to an inhibition in melatonin induced ROS production^39^, suggesting that H_2_O_2_ might act downstream of Ca^2+^ in melatonin-mediated cold tolerance.

Cold-induced secondary signals further activate different transcriptional regulators (such as *BHLH, inducer of CBF expression 1 (ICE 1*), *C-repeat-binding factor (CBF*), *WRKY*, and *MYB*) via activation of phosphoprotein kinases, such as CDPKs and MAPKs^1^,^3^. Eventually, cryo-protective compounds (such as late embryogenesis abundant (LEA) proteins, molecular chaperones, detoxification enzymes) are induced to maintain normal physiological processes^4^. Recent studies revealed that melatonin can activate cold defense systems by regulating the expression of some transcriptional regulators (such as *ZAT10, ZAT12, CBFs*) and COR genes (such as *COR15a*) encoding major cryoprotective proteins^15^,^16^. Likewise, in the present study, we found melatonin increased the expression of an abundance of transcription factors (i.e. *BZIP, BHLH, WRKY, MYB*, and *HSF*) and genes that encode cryo-protective compounds (i.e. LEA-HRGP, HSPs, peroxidase, and lipoxygenase) during cold stress. These results suggest that a set of transcriptional regulators and genes encoding cryo-protective compounds are involved in melatonin-mediated cold tolerance (Table 2).

The modes of cold stimulus transduction to the nucleus and upstream regulatory modules that govern nuclear events allowing the TFs to directly control *COR* gene expression remain largely unspecified. However, in recent years, accumulating data suggests that plant hormones function as governors, additionally governing other signal events, such as secondary signaling induction and protein phosphorylation in responses to cold stress^5^,^40^,^41^,^42^. Moreover, interactions between melatonin and plant hormones have been reported. Melatonin not only regulates endogenous contents of hormones (such as auxin, ABA, GA, and ET), but also affects the expression of most genes in ABA, salicylic acid (SA), JA, and ET pathways^43^. Furthermore, we observed that exogenous melatonin application alters the expression of a set of genes involved in various hormone-signaling pathways (Table 3). The expression of most genes involved in ET, GA, Auxin, and JA pathways were up-regulated by melatonin, but consistently with previous studies, the transcription of ABA receptor *PYL8* was down-regulated by melatonin under cold stress^44^,^45^. Therefore, various plant hormones might be involved in melatonin-mediated cold tolerance.

In conclusion, our study shows that local application of melatonin on leaves or roots not only induces tolerance to cold stress in the site of application, but also leads to systemic induction of cold tolerance in untreated distant roots or leaves, respectively. As a potent long-distance signal, melatonin might be transported from treated to distant untreated tissues via vascular bundles. We found melatonin-induced cold tolerance in distant organs to be associated with the induction of the antioxidant system. Moreover, analysis of high-throughput mRNA sequencing revealed that abundance of cold defense-related genes involved in signal sensing as well as transduction, transcriptional regulation, protection and detoxification, and hormone signaling might be involved in melatonin-mediated cold tolerance (Fig. 7).

## Methods

### Plant materials

Watermelon (*Citrullus lanatus* L., cv. Y134) seeds were sown directly in pots filled with a mixture of peat/vermiculite (3/1, v/v) after surface sterilization with 5% sodium hypochlorite (NaOCl) solution. The seedlings were grown in growth chambers with the following environmental conditions: a constant relative humidity of 70–80%, a 12-h photoperiod, 25/18 °C (day/night), and a photosynthetic photon flux density (PPFD) of 600 μmol m^−2^ s^−1^. The plants were watered daily and fertilized with Hoagland’s nutrition solution at one day interval. Upon the appearance of the first true fully expanded leaves, a group of eight seedlings was transplanted into a container (40 × 25 × 15 cm) filled with Hoagland’s nutrient solution.

### Experiment 1

To examine the effect of foliar or rhizospheric application with melatonin on local tolerance to cold stress, watermelon plants at the four-leaf-stage were sprayed with 50, 150, 300, 500, or 800 μM melatonin (20 mL per plant) on leaves (LMT), or their roots were treated with 0.05, 0.15, 1.5, 15, or 50 μM melatonin (RMT) by adding required amount of stock solution of melatonin into the culture solution. Each application was repeated thrice (once a day). Treatments that did not receive melatonin were simultaneously sprayed with distilled water containing the same ratio of ethanol. Melatonin (acquired from Sigma-Aldrich, St. Louis, MO, USA) solutions were prepared by dissolving the solute in ethanol followed by dilution with Milli-Q water (ethanol/water (v/v) = 1/10,000). Twelve hours after the 3^rd^ application, plants with foliar or rhizospheric melatonin treatment were exposed to either aerial cold at 4 °C (SC) or root-zone cold at 10 °C (RC) for 72 h, respectively. For the SC stress treatment, seedlings were transferred to a chamber maintained at 4 °C, while the nutrient solution was heated to and maintained at approximately 25 °C using a heating rod with a temperature controller. For the RC stress treatment, the nutrient solution of seedlings was cooled to and maintained at approximately 10 °C using frozen ice bags, while the seedlings were kept in a chamber maintained at 25 °C. Samples of leaf (the second fully expanded leaf beneath the growing point) and root (the length of about two third of the roots below the root tips, except main roots) were taken at 72 h to analyze their cold tolerance. A 150-μM concentration of melatonin for leaf or a 1.5-μM concentration of melatonin for root was used for the rest of the experiments.

### Experiment 2

To determine whether melatonin could induce cold tolerance in distant organs, the plants with RMT (1.5 μM) or LMT (150 μM) treatment were exposed to SC or RC stress for 72 h, respectively. Leaf samples in plants with RMT and, or SC treatments and root samples in plants with LMT and, or RC treatments were collected at 24 h to analyze transcript levels of cold response genes, at 24 h and 72 h to measure melatonin contents, antioxidant enzyme activities, and stress tolerance. Additionally, xylem sap exudates of plants with RMT and, or SC treatments were collected at 24 h and the melatonin exudation rate was analyzed to confirm potential transport of melatonin via vascular bundles.

### Experiment 3

To determine melatonin effects on the defense gene network in response to cold stress, watermelon seedlings at the four-leaf stage were sprayed with 150 μM melatonin for three times (once a day). Twelve hours after the 3^rd^ spray of melatonin, whole plants were exposed to a 4 °C cold stress for 36 h with a 12-h photoperiod and PPDF of 600 μmol m^−2^ s^−1^. Leaf samples were taken at 6 h after exposure of plants to cold stress to perform high-throughput mRNA sequencing with two biological replicates for each treatment.

### Analysis of gas exchange, chlorophyll a content, and root vitality

Net photosynthetic rate (Pn), was measured on the second fully expanded leaf beneath the growing point using a LI-6400 portable photosynthesis system (Li-6400; Li-Cor, Lincoln, NE, USA) equipped with an LED red/blue light source (6400-02B). The measurement was performed maintaining the air temperature, relative humidity, CO_2_ concentration, and PPFD at 25 °C, 80%, 380 μmol mol^−1^, and 600 μmol m^−2^ s^−1^, respectively. According to the method of Arnon^46^, leaf chlorophyll a (Chl a) was extracted in 80% acetone and Chl a contents were determined colorimetrically using a spectrophotometer (UV6100, Shanghai, China).

Root vitality was determined according to the method of Clemensson-lindell^47^. The fresh roots (0.3 g) were cut into small pieces 1- to 2-mm long, and were incubated with 6 ml of 0.6% (w/v) TTC in 0.06 M Na_2_HPO_4_–KH_2_PO_4_ at 37 °C for 3 h. After addition of 0.05% (v/v) Tween 20, the samples were vacuum-infiltered for 15 min. After incubation, the root pieces were washed twice with 5 mL of distilled water. Then, the samples were extracted in 95% (v/v) ethanol at 80 °C for 15 min and absorption at 520 nm was measured.

### Melatonin measurements and xylem sap collection

Melatonin was extracted using the acetone-methanol method as described by Pape and Lüning^48^ and measured by enzyme-linked immunosorbent assay following the method of Okazaki and Ezura^49^. Briefly, 1 g of frozen leaf or root samples were extracted in 10 mL of extraction mixture (acetone:methanol:water at 89:10:1). The extract was centrifuged (4,500 g, 4 °C) for 5 min and the supernatant was added 2 mL of 1% trichloric acid for protein precipitation. The extraction was repeated twice by resuspending the residues and the combined supernatants were concentrated in a vacuum concentrator to a total volume of 10 mL and then adjusted with distilled water to 50 mL. After centrifugation (10,000 g, 10 °C) for 15 min, the supernatants were filtered through Sep-Pak C18 cartridge (Millipore, Milford, MA, USA) for the purification of melatonin. Melatonin was measured using an immunoassay kit (Shanghai Lanpai Biotechnology Co., Ltd, Shanghai, China) following the manufacturer’s instructions. The result was quantified based on a standard curve of known melatonin concentrations. Colorimetric recording was carried out via Multimode Plate Reader M200 pro (Tecan, Männedorf. Schweiz).

Xylem sap exudates were collected by cutting the stem 5 cm above the soil with a sterile razor blade and the cut surface was blotted for several times, with sterile filter paper. Xylem sap exuded thereafter was collected using sterile micropipette tips (20 μL).

### Analysis of H_2_O_2_, O_2_·^−^, and malondialdehyde

H_2_O_2_ was extracted from 0.5 g leaf or root samples in 3 mL of 1 M HClO_4_ at 4 °C, and then the homogenate was centrifuged at 6,000 g for 5 min at 4 °C. The supernatant was adjusted to pH 6.0–7.0 with 4 M KOH and filtered through an AG1 × 8 prepacked column (Bio-Rad, Hercules, CA, USA). After eluted with 4 mL double-distilled H_2_O, aliquot of sample (800 μL) was mixed with 400 μL reaction buffer containing 4 mM 2,2′-azino-di (3-ethylbenzthiazoline-6-sulfonic acid) and 100 mM potassium acetate at pH 4.4, 400 μL deionized water and 0.25 U of horseradish peroxidase. H_2_O_2_ content was measured at OD412^50^.

Superoxide production was quantified according to the method of Elstner and Heupel^51^ with a slight modification. 0.5 g of samples were homogenized with 3 mL of 65 mM potassium phosphate buffer (pH 7.8) and centrifuged at 5,000 g for 10 min. The incubation mixture contained 1 mL of the supernatant, 0.9 mL of 65 mM phosphate buffer (pH 7.8), and 0.1 mL of 10 mM hydroxylamine hydrochloride. After incubation at 25 °C for 20 min, 17 mM sulfanilamide and 7 mM α-naphthylamine were added to the incubation mixture. After reaction at 25 °C for 20 min, ethyl ether in the same volume was added and centrifuged at 1,500 g for 5 min. The absorbance in the aqueous solution was read at 530 nm. Sodium nitrite was used as a standard solution to calculate the production rate of superoxide.

Malondialdehyde (MDA) as an end product of lipid peroxidation was measured by 2-thiobarbituric acid (TBA) reaction according to the method of Hodges^52^. Briefly, leaf or root samples (0.3 g) were homogenized in 5 mL of 10% (w/v) trichloroacetic acid (TCA). The homogenate was centrifuged at 3,000 g for 10 min and 4 ml of 20% TCA containing 0.65% (w/v) TBA was added to 1 mL of supernatant. The mixture was heated at 95 °C for 25 min and immediately cooled to stop the reaction. Then those samples were centrifuged at 3,000 g for 10 min and the absorbance of the supernatant was recorded at 440 nm, 532 nm, and 600 nm. The assays of H_2_O_2_, O_2_·^−^, and MDA were performed using a spectrophotometer (UV6100, Shanghai, China).

### Antioxidant enzyme extraction and activity assay

Leaf or root samples (0.5 g each) were ground with 3 mL ice-cold 25 mM HEPES buffer (pH 7.8) containing 0.2 mM EDTA, 2 mM AsA, and 2% PVP. The homogenates were centrifuged at 4 °C for 20 min at 12,000 g, and the resulting supernatants were used for the determination of enzymatic activity by using spectrophotometric methods. SOD activity was assayed by following the Stewart and Bewley^53^ method based on photochemical reduction of NBT. POD was assayed by recording the changes in absorbance at 470 nm caused by guaiacol oxidation following the procedure described by Cakmak and Marschner^54^. CAT activity was measured as a decline in A_240_ using the method of Patra^55^.

### RNA extraction and qRT-PCR analysis

Total RNA was extracted using an RNA extraction kit (Axgen, Union City, CA, USA) according to the manufacturer’s instructions. Residual DNA was removed with DNase Mini Kit (Qiagen, Hilden, Germany). One microgram of total RNA was used for reverse transcription using the ReverTra Ace qPCR RT Kit (Toyobo, Osaka, Japan) following the manufacturer’s instructions. The gene-specific primers for qRT-PCR were designed on the basis of cDNA sequences as shown in Supplemental Table S1 and watermelon *β-actin* gene was used as an internal control^56^. The qRT-PCR assay was performed using an iCycler Iq TM Multicolor PCR Detection System (Bio-Rad, Hercules, CA, USA). PCR products were amplified using the Premix ExTaq II (2×) Kit (Takara, Tokyo, Japan). The PCR conditions consisted of denaturation at 95 °C for 3 min, followed by 40 cycles of denaturation at 95 °C for 30 s, annealing at 58 °C for 30 s and extension at 72 °C for 30 s. The quantification of mRNA levels was performed according to the method of Livak and Schmittgen^57^.

### RNA-seq library construction and sequencing

Plants underwent one of four treatments: Control (CK), Melatonin (MT), Cold, and Melatonin + Cold (MT-C). Eight independent mRNA libraries with two biological replicates for each treatment were sequenced by a service provider Gene Denovo Co. (Guangzhou, China). After total RNA was extracted using the RNA extraction kit (Promega, USA), mRNA was enriched by oligo(dT) beads and then cleaved into short fragments using fragmentation buffer and reverse-transcribed into cDNA with random primers. Second-strand cDNA were synthesized by DNA polymerase I, RNase H, dNTP and buffer. Then the cDNA fragments were purified with QiaQuick PCR extraction kit, end repaired, poly (A) added, and ligated to Illumina sequencing adapter. The ligation products were size selected by agarose gel electrophoresis; PCR amplified, and sequenced using Illumina HiSeq^TM^ 2500. The sequences have been deposited into the NCBI Sequence Read Archive database (SRP078211, SRA438977).

### Analysis of Illumina sequencing results

Raw reads were filtered to remove low quality sequences and the obtained clean reads were mapped to the watermelon reference genome, allowing up to one mismatch. Unigenes mapped by at least one read, in at least one sample, were identified for further analysis. The expression level of each unigene was calculated and normalized to generate FPKM (fragments per kilobase of exon per million mapped fragments). The false discovery rate (FDR) was used to determine the threshold of the P-value in multiple tests. To identify genes regulated by melatonin or/and cold stress in watermelon leaves, we analyzed the differentially expressed genes (DEGs) in comparison of MT/CK, Cold/CK, MT-C/CK, and MT-C/Cold. A combination of the absolute value of log_2_ Ratio ≥2 and the false discovery rate (FDR) ≤0.05 were used as the threshold to judge the significance of gene expression difference^58^. To determine the main biological functions, we subjected the DEGs to Gene Ontology (GO) classification based on their involvement in process in the Cucurbit Genomics Database (http://www.icugi.org) with watermelon 97103 v1.

### Statistical analysis

The experiment was a completely randomized design with three replications. Each replicate contained at least 12 plants. Analysis of variance (ANOVA) was used to test for significance, and significant differences (*P* < 0.05) between treatments were determined using Tukey’s test.

## Additional Information

**How to cite this article**: Li, H. *et al*. Local melatonin application induces cold tolerance in distant organs of *Citrullus lanatus* L. via long distance transport. *Sci. Rep.* **7**, 40858; doi: 10.1038/srep40858 (2017).

**Publisher's note:** Springer Nature remains neutral with regard to jurisdictional claims in published maps and institutional affiliations.

## Supplementary Material

Supplementary Table and Figure

## Figures and Tables

**Table 1:** Receptor and secondary signaling-related gene expression of watermelon leaves influenced by melatonin and, or cold stress.

Gene ID	MT/CK Log_2_FC	Cold/CK Log_2_FC	MT-C/CK Log_2_FC	MT-C/Cold Log_2_FC	Description
**Receptor**					
Cla000464	−0.32	0.91	2.27*	1.35	Receptor-like kinase
Cla014506	−0.24	−3.16*	−2.08*	1.08	Receptor-like kinase
Cla003448	0.38	−3.55*	−1.23	2.32	Receptor-like protein kinase
Cla022940	0.08	−10.46*	−1.63	8.83	Receptor-like protein kinase
Cla019085	1.24	2.37*	1.62	−0.74	Receptor-like protein kinase
Cla002252	−0.01	−2.20*	−0.64	1.56	Receptor protein kinase-like protein
Cla002608	0.04	−2.30*	−1.15	1.15	Receptor protein kinase-like protein
Cla003590	−0.01	−1.11	−2.90*	−1.79	Receptor protein kinase-like protein
Cla003671	−2.15	1.47	2.16*	0.69	Receptor protein kinase-like protein
Cla006279	0.17	0.88	−1.93	−2.80*	Receptor protein kinase-like protein
Cla012725	0.40	−2.46*	−1.32	1.14	Leucine-rich repeat (LRR) receptor like protein kinase
Cla014545	0.39	−1.44	−2.46*	−1.02	LRR receptor-like protein kinase
Cla004300	−0.39	−1.43	−2.49*	−1.06	LRR receptor-like tyrosine-protein kinase
Cla022876	0.15	−3.43*	−2.38*	1.05	LRR receptor-like kinase
Cla010135	−0.22	−0.21	1.94	2.15*	LRR receptor-like protein kinase
Cla010175	−1.66	−3.62*	−0.68	2.94	Receptor expression-enhancing protein 5
Cla012436	−0.42	−10.04*	−2.36*	7.68	Receptor lectin protein kinase-like
Cla015719	−0.22	1.19	2.61*	1.42	Lectin receptor kinase 1
Cla017931	2.21	3.80*	5.23*	1.43	B-lectin receptor kinase
Cla020000	−0.19	−2.97*	−1.53	1.44	TGF-beta receptor
Cla020646	−0.34	−4.22*	−1.56	2.66	SPla/RYanodine receptor SPRY
Cla021503	−1.89	−2.34*	−1.11	1.24	Xenotropic and polytropic retrovirus receptor 1
Cla023335	0.17	−1.91	−2.96*	−1.05	G-type lectin S-receptor-like serine/threonine-protein kinase
**Secondary signalling**					
Cla008583	0.01	−4.16*	−2.27	1.89	Calcium dependent protein kinase 25
Cla021259	0.00	11.65	13.05*	1.41	Calcium-dependent membrane targeting
Cla011195	1.40	1.56	2.42*	0.86	Calmodulin-binding protein
Cla020869	−0.12	−4.28*	−0.47	3.81	Calmodulin binding protein
Cla017404	0.16	−2.84*	−1.78	1.06	Sodium-calcium exchanger 3
Cla014031	0.11	1.68	2.85*	1.17	Plasma membrane calcium-transporting ATPase 3
Cla018105	0.11	1.35	2.63*	1.28	Calcium/proton exchanger
Cla017196	0.34	0.87	2.02*	1.15	Respiratory burst oxidase-like protein
Cla015949	−0.55	1.10	−1.01	−2.11*	Membrane transporter D1
Cla008163	0.06	−0.12	−2.37*	−2.25	1-phosphatidylinositol-4
Cla006310	−0.12	−1.79	−2.96*	−1.16	5-bisphosphate phosphodiesterase Phospholipase A1
Cla012695	−1.97	2.15*	1.21	−0.95	Acyl-protein thioesterase 2
Cla009398	0.44	2.37*	0.49	−1.89	Membrane transporter D1
Cla018548	−0.47	−2.00**	−1.02	0.98	COBRA-like protein

Watermelon seedlings at the four-leaf stage were treated as described in Fig. 6. Data shown are the log2 fold-changes values (Log_2_FC) for genes in comparison of MT/CK, Cold/CK, MT-C/CK, and MT-C/Cold. *Indicates significant difference.

**Table 2:** Transcription factor and defense-related gene expression of watermelon leaves influenced by melatonin and, or cold stress.

Gene ID	MT/CK Log_2_FC	Cold/CK Log_2_FC	MT-C/CK Log_2_FC	MT-C/Cold Log_2_FC	Description
**Transcription factors**					
Cla023484	−0.46	1.53	2.44*	0.91	BZIP transcription factor protein
Cla015627	−0.26	−2.63*	−0.30	2.32*	BZIP transcription factor family protein
Cla014048	−8.65	−2.03	−2.78*	−0.74	BZIP transcription factor family protein
Cla020795	−0.95	−1.89	−2.84*	−0.95	BZIP transcription factor
Cla021776	−0.98	2.50	3.09*	0.60	MYB transcription factor
Cla010413	7.90	7.13	8.24*	1.11	MYB transcription factor
Cla019999	−0.37	−2.10*	−0.99	1.11	MYB transcription factor
Cla010316	0.25	−3.07*	−1.59	1.48	MYB transcription factor
Cla005982	1.53	3.30	3.78*	0.48	MYB-related transcription factor
Cla017441	−0.34	−3.08*	−1.66	1.42	MYB family transcription factor-like
Cla020715	1.20	4.05*	3.36	−0.69	MYB family transcription factor-like
Cla017337	−0.14	2.85*	3.95*	1.09	MYB-like transcription factor Myb 5
Cla023007	−0.35	−2.79*	−1.46	1.32	MYB-like protein
Cla021148	0.26	1.17	2.13*	0.97	MYC2 transcription factor
Cla007867	0.10	−3.15*	−2.07*	1.09	MYC2 transcription factor
Cla010890	1.48	0.34	2.10*	1.76	BHLH transcription factor
Cla006061	−1.00	−2.40*	−1.28	1.12	BHLH transcription factor
Cla010576	−0.43	−2.78*	−1.49	1.29	BHLH transcription factor
Cla022767	0.88	1.54	2.00*	0.46	BHLH family protein
Cla009965	0.49	−1.18	1.54	2.72*	BHLH family protein
Cla018968	−2.01	−2.47*	−1.80	0.67	BHLH family protein
Cla018502	−2.27	−7.64*	−0.67	6.96	BHLH family protein
Cla021067	−0.34	−2.85*	−1.08	1.77	WRKY family transcription factor
Cla017213	0.41	3.81*	4.81*	1.01	WRKY transcription factor 4
Cla000713	0.45	1.79	2.32*	0.53	Heat stress transcription factor
Cla014794	1.88	2.15	3.41*	1.26	Heat stress transcription factor A3
Cla022583	0.24	−2.32*	−1.02	1.30	Heat stress transcription factor
Cla021592	0.09	−3.75*	−2.32*	1.43	Heat stress transcription factor A3
**Defense genes**					
Cla010028	0.73	−2.50*	−0.65	1.85	Late embryogenesis abundant (LEA) hydroxyproline-rich glycoprotein
Cla012714	2.38	2.90	3.86*	0.96	Heat shock protein 20
Cla006400	−0.13	3.68*	4.84*	1.16	Heat shock protein DnaJ
Cla014224	−0.22	−4.07*	−1.84	2.23	Heat shock protein DnaJ
Cla019553	−0.64	−5.48*	−2.35*	3.13	Heat shock protein DnaJ
Cla003912	−0.11	−4.07*	−2.89*	1.18	Heat shock protein dnaJ
Cla021766	−0.21	−4.30*	−1.92	2.38	Heat shock protein dnaJ 49
Cla021632	0.03	−1.83	−2.11*	−0.28	Heat shock protein 70
Cla017643	−0.99	−3.52*	−1.85	1.67	Heat shock protein 101
Cla013681	0.03	−2.28*	−0.45	1.84	Heat shock-like protein
Cla018862	−0.81	−2.03*	−0.79	1.24	Heat shock protein-related
Cla003190	0.29	−2.74*	−0.24	2.50*	Peroxidase
Cla010497	−0.38	−1.37	0.65	2.01*	Peroxidase a
Cla003194	1.94	2.55*	1.52	−1.03	Peroxidase
Cla020908	−0.80	−2.88*	−1.22	1.66	Peroxidase
Cla003187	−0.08	−0.89	−2.25*	−1.35	Peroxidase
Cla019901	0.70	−0.70	2.66*	3.36	Lipoxygenase
Cla019905	0.10	−0.73	1.52	2.25*	Lipoxygenase
Cla019899	−0.04	−2.81*	−4.72*	−1.91	Lipoxygenase

Watermelon seedlings at the four-leaf stage were treated as described for Fig. 6. Data shown are the log2 fold-changes values (Log_2_FC) for genes in comparison of MT/CK, Cold/CK, MT-C/CK, and MT-C/Cold. *Indicates significant difference.

**Table 3:** Plant hormone signal-related gene expression of watermelon leaves influenced either by melatonin and, or cold stress.

Gene ID	MT/CK Log_2_FC	ColdCK Log_2_FC	MT-C/CK Log_2_FC	MT-C/Cold Log_2_FC	Description
**Ethylene**					
Cla002237	0.32	1.91	2.89*	0.98	ERF transcription factor
Cla021069	5.82	8.02	9.88*	1.85	ERF transcription factor 1b
Cla021070	0.94	1.76	4.87*	3.11*	ERF transcription factor 1b
Cla007092	0.21	−1.26	−2.27*	−1.01	ERF transcription factor 2a
Cla013573	−0.47	−10.48*	−1.47	9.01	ERF transcription factor 2b
Cla017389	0.17	3.50	5.45*	1.95	ERF transcription factor 2b
Cla022212	0.12	−3.90*	−2.08*	1.81	ERF transcription factor 4
Cla016785	−0.47	−3.07*	−1.58	1.49	ERF transcription factor 5
Cla014051	−1.03	−2.94*	−0.90	2.04	AP2-like ERF transcription factor
Cla022648	−0.71	−4.40*	0.19	4.59*	AP2-like ERF transcription factor
**Gibberellin**					
Cla007770	−1.49	2.32	2.47*	0.15	D-limonene synthase
Cla005397	1.03	0.17	2.29*	2.12*	Gibberellin 2-oxidase
Cla022611	0.31	0.85	3.14*	2.29	Gibberellin-regulated family protein
Cla007779	−0.48	2.87*	1.51	−1.36	Limonene synthase
Cla020559	0.00	−2.02*	−0.43	1.59	GATA transcription factor 8
**Auxin**					
Cla010118	0.78	−1.91	−3.21*	−1.30	IAA-amino acid hydrolase
Cla015856	−12.07	1.93	2.52*	0.59	Auxin-induced SAUR-like protein
Cla016616	1.56	1.42	2.23*	0.81	SAUR-like auxin-responsive protein
Cla006581	−0.65	−2.11*	−0.97	1.14	Auxin transporter-like protein 1
Cla009105	0.19	−2.01*	−1.68	0.33	Auxin response factor 8-1
Cla011242	−0.10	−2.48*	−1.14	1.34	Iaa-amino acid hydrolase 11
Cla014809	−1.04	−2.23*	−1.04	1.20	Auxin responsive protein
Cla015002	−0.26	−3.01*	−1.90	1.11	Auxin response factor 9
Cla019806	−0.56	−2.42*	−1.23	1.19	Auxin responsive protein
**Abscisic acid**					
Cla004904	0.16	−2.29	−12.67*	−10.38	Abscisic acid receptor PYL8
**Cytokinins**					
Cla006831	−1.09	−0.87	−2.66*	−1.78	Cytokinin oxidase/dehydrogenase
Cla013090	−0.40	−1.96	0.66	2.61*	Cyclin D3-1
Cla013975	−0.07	−2.66*	−1.41	1.25	Cyclin D
**Jasmonic acid**					
Cla011143	−0.39	1.68	2.79*	1.11	Jasmonate ZIM-domain protein 3
Cla012536	−0.98	3.50*	5.17*	1.67	Protein TIFY 7

Watermelon seedlings at the four-leaf stage were treated as described for Fig. 6. Data shown are the log2 fold-changes values (Log_2_FC) for genes in comparison of MT/CK, Cold/CK, MT-C/CK, and MT-C/Cold. *Indicates significant difference.
