# Supplementary material for: Local melatonin application induces cold tolerance in distant organs of Citrullus lanatus L. via long distance transport
Source: Sci Rep. 2017 Jan 19;7:40858. doi: 10.1038/srep40858 (PMC5244382; doi:10.1038/srep40858)

Title:

Local melatonin application induces cold tolerance in distant organs of *Citrullus lanatus* L. via long distance transport

Authors:

Hao Li[1†], Jingjing Chang[1†], Junxian Zheng[1], Yuchuan Dong[1], Qiyan Liu[1], Xiaozhen Yang[1], Chunhua Wei[1], Yong Zhang[1], Jianxiang Ma[1] & Xian Zhang[1*]

[1]*Department of Horticulture, Northwest A&F University, Taicheng Road 3, Yangling 712100, Shaanxi, P.R. China*

[†]*These authors contributed equally to this work.*

[*] *To whom correspondence should be addressed. Email: zhangxian098@126.com*

Table S1. Primers used for qRT-PCR assays. F indicates forward and R indicates reverse.

Gene	Primer pairs (5’-3’)
β-actin (*Cla007792*)	F: CCATGTATGTTGCCATCCAG
	R: GGATAGCATGGGGTAGAGCA
CDPK18 (*Cla014285*)	F: GCCATGAGAATGTGGTTCAG
	R: CAGCTGCATCTTTCTCGGTA
MAPK16 (*Cla009366*)	F: AGGCCACAAGTGGATAGGAC
	R: GCAACACAGAGCCAACAACT
RBOH (*Cla011409*)	F: CTTGCCAAGGATGGGTATCT
	R: TTCGACTCAAAGCATCGAAC
RBOH-like (*Cla017196*)	F: AAATCACCCTCGATATTCGC
	R: TGCTCGGAAAGAGGAAGATT
ERF (*Cla002237*)	F: GGTTTCCAAGGAGAAATCCA
	R: TGCCATTGTTGTTGTTGTTG
BZIP (*Cla023484*)	F: AACAATGGAGGACGAAGGTC
	R: TCGCATCCAACATACTTGGT
Myb-like (*Cla017337*)	F: GGAAGAACCGACAACGAAAT
	R: ATTCCAGCCTCATAGCGTCT
BHLH (*Cla010890*)	F: CATCTGTTCTTCTGGAGGCA
	R: GACATAGGCCTCTGCTCCTC
WRKY4 (*Cla017213*)	F: CCTCCAACCCAAGATTTGAC
	R: TTCGTTAAGGAAGAGGCCAT
HSF (*Cla000713*)	F: CCCTTCCTCACCAAGACCTA
	R: ATGAAGGTAGAGCCCGATTG

Table S2. Reads abundance of various classifications of mRNAs in control plants (CK) and plants treated with melatonin (MT), Cold, and melatonin+cold (MT-C).

Sample Name	Raw Reads Num	Clean Reads Num	rRNA	Unique Mapped Reads	Multiple Mapped reads	Kown Gene Num	New Gene Num	All Gene Num
CK-1	40326590	39919518	5534	36775488	490772	16484	492	16976
CK-2	38043848	37592838	4218	34451398	403064	16341	494	16385
MT-1	37704638	37288558	3796	34117719	471554	16377	490	16876
MT-2	36489514	36068804	3808	33058872	517732	16321	492	16813
Cold-1	36122786	35809268	7762	32579557	651900	16216	476	16692
Cold-2	35953956	35601736	4852	32616553	379044	16068	486	16554
MT-C-1	32845006	32532788	3890	29881207	383710	16146	479	16625
MT-C -2	33262622	30835220	6650	28069430	429846	16234	479	16713

Figure S1. The correlation of gene expression between two biological repeats in melatonin and, or cold treatments.

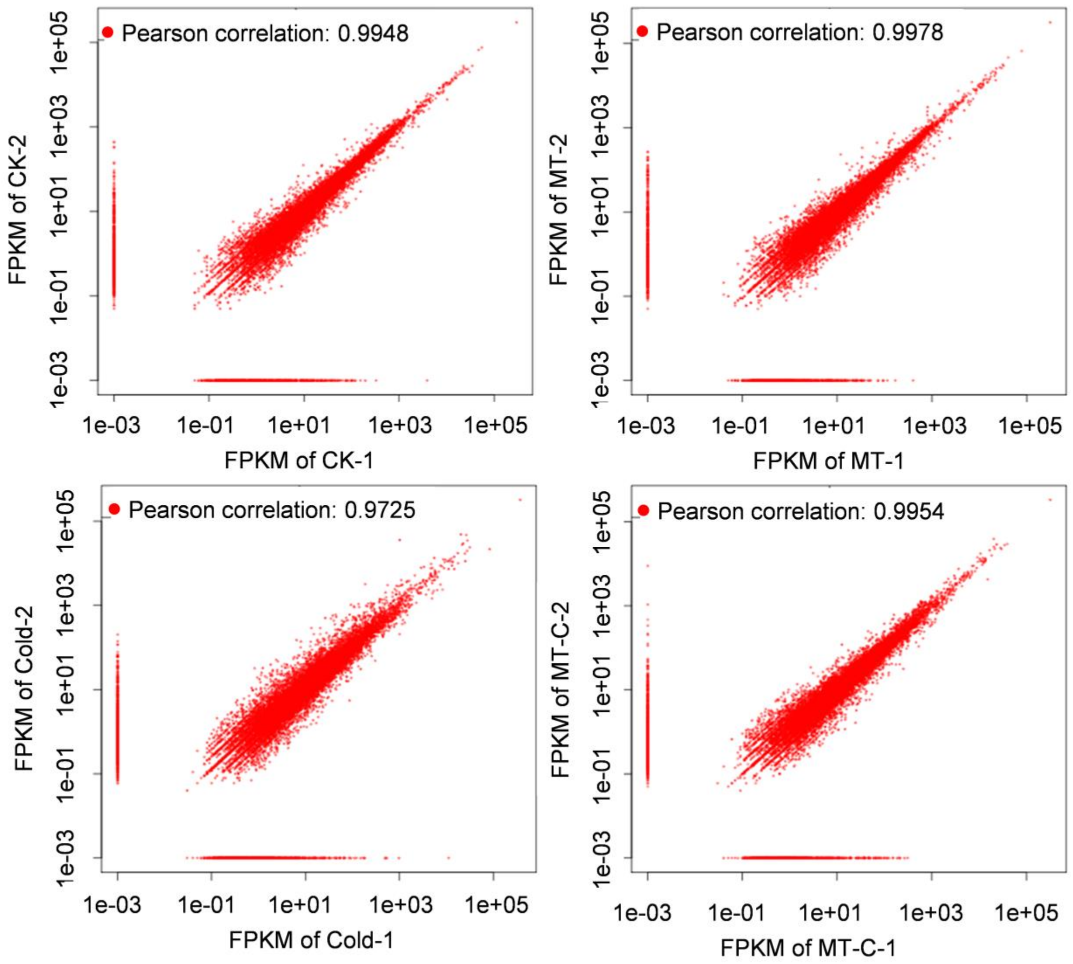

Supplement: Supplementary Table and Figure [file srep40858-s1.pdf]
